# The effect of aromatherapy with Lavender-Neroli oil and music in management of pediatric dental anxiety: a randomized control trial

**DOI:** 10.1038/s41405-024-00186-8

**Published:** 2024-01-29

**Authors:** Rama Abdalhai, Chaza Kouchaji, Rasha Alkhatib

**Affiliations:** 1 Department of Pediatric Dentistry, College of Dentistry, Damascus University, Damascus, Syria; 2 Department of Pharmacognosy, College of Pharmacology, Damascus University, Damascus, Syria

**Keywords:** Oral analgesics, Paediatric dentistry, Dental anaesthesia

## Abstract

**Aim:**

This study aimed to evaluate the efficacy of aromatherapy with Lavender-Neroli essential oil combined with background music in reducing dental anxiety and pain during anesthesia in children.

**Materials and methods:**

A total of 56 children aged 6–10 years old who needed dental treatment with inferior alveolar nerve injection (IANB) were randomly divided into two groups: Group 1 (Experimental, *n* = 28) aromatherapy with music group, and Group 2 (Control, *n* = 28) the placebo group. Children in the group 1 were asked to inhale the aromatic blend of Lavender-Neroli essential oil using a nasal mask similar to one that is used for nitrous oxide after modifying it by adding a 3D printed box on its circle hole and listening to their favorite music as a background before 5 min and during anesthesia, meanwhile in the placebo group children were asked to wear an empty nasal mask. Anxiety and pain were been assessed before and after anesthesia using the self-report anxiety scale Facial image scale (FIS), Observational pain assessment scale Face-Legs-Activity-Cry-Consolability (FLACC), heart rate, SPO2 saturation, diastolic and systolic blood pressure.

**Results:**

Dental anxiety and vital signs except SPO2 saturation were significantly lower in the aromatherapy with music group when compared to the control group (*p* < 0.05), with no differences in pain perception between groups (*p* = 0.176).

**Conclusions:**

Aromatherapy with Lavender-Neroli oil combined with music seems to be a useful and safe non-pharmacologic technique for managing dental anxiety in children.

## Introduction

Dental fear and anxiety (DFA) is the term that is used to describe sorts of negative feelings shown by patients towards dental treatment, and it continues to be one of the most source of challenges for dentists when providing dental care to children [1, 2]. About one-tenth to one-fifth of children suffer from dental anxiety as its prevalence in children ranges between 10 and 23.9% [3, 4]. The dental anxious patient tends to avoid necessary treatments, which affect oral health and its related quality of life negatively [5, 6].

For the dentist, treating a fearful child seems to be harder and requires more time due to less cooperation and behavior problems [3, 7]. The treatment is considered successful when the dentist can manage the child’s fears and lead him/her toward a positive behavior [6]. In dental treatment, many anxiety sensory triggers can be found including the sight of the needle shocking, the sound and sight of the drill, and the smell of dental materials (eugenol, dentine cut, other) [1, 8]. Therefore, techniques that help to reduce sensory stimulus in the dental clinic and make it less dangerous can be useful in managing children’s anxiety toward dental treatment, this includes aromatherapy and music [9].

Aromatherapy is one of the Complementary alternative medicines that use plant’s Secondary metabolism called essential oils via inhalation, oral, and topical application to obtain therapeutic benefits including anxiolytic, analgesic, antidepressant, and rejuvenation of the human body [10, 11]. The use of aromatic oils in the healing process goes back to Egyptian and Chinese culture [12].

Recently, several studies have shown the effectiveness of aromatherapy via inhalation as a non-pharmacological method with minimal side effects, low costs, and a simple way to reduce anxiety in medical [13, 14] and dental settings [15–17]. As the Aroma from essential oils can affect our sense of smell which has inputs in the limbic system the area of the brain related to developing emotion, memory, and hormone secretion [12].

Two of the most common essential oils that are widely used in aromatherapy are Lavender and Citrus essential oils. Lavender oil is the essential oil extracted by hydrodistillation of Lavandula angustifolia flowers with anxiolytic and analgesic effects [18, 19]. It belongs to the Lamiaceae family with a great component of linalool and linalyl acetate which play an important role in lavender’s anxiolytic properties as they act like a sedative [11, 19].

One of the citrus essential oils that is used in aromatherapy to reduce anxiety and pain during stressful medical conditions is Neroli [20, 21]. Neroli is the essential oil extracted by hydrodistillation of Citrus arauntium flowers which belongs to the Rutaceae family [21, 22]. It has shown anxiolytic effects due to its major component of monoterpenes which have an impact on the nervous system such as Linalool and limonene [22].

Music is one of the effective, non-invasive, and non-pharmacological techniques that has attracted attention for its effect in reducing dental anxiety [23, 24]. Music induces an anxiolytic effect and improves mental skills as it can stimulate the relaxation brain’s waves such as alpha, beta, and theta, and increase the functional connection between the brain’s regions [25].

In pediatric dentistry, only two studies evaluated the effectiveness of the combined use of aromatherapy and music in reducing children’s dental anxiety during fissure sealant which is considered non-invasive procedure [9, 26], and there is no study evaluating the use of the two techniques together in reducing pediatric dental anxiety during invasive dental procedures such as anesthesia injection, therefore this study aimed to investigate the effect of aromatherapy combined with music in reducing dental anxiety and pain during Inferior alveolar nerve block (IANB) injection in children.

## Materials and methods

### Study design and ethical considerations

This was a single blind-randomized control trial with a two-arm parallel superiority design with a 1:1 allocation which was conducted during the period between October 2021 to October 2023 in the Department of Pediatric Dentistry at the Faculty of Dentistry, Damascus University, Syria.

All procedures in this study were in accordance with the declaration of Helsinki guidelines and its Consort recommendations and received ethical approval from the Local Research Ethics Committee of the Faculty of Dentistry [Approval No. UDDS-502-220420121/SRC-3183]. The research was registered on clinicaltrials.gov [NCT05759286] and the informed consent was taken from the Participant’s parents or legal guardians before the procedures were carried out and after an adequate explanation was provided about this study and the procedures that will be done.

### Sample and sampling methods

The sample size was calculated using G* Power 3.1.9.4 [Heinrich-Heine-Universität, Düsseldorf, Germany], based on the effect size of the previous study with pulse rate’s Means ± SD in control and experimental groups were 10.8 ± 7.5, −8.3 ± 6.8, respectively [26]. The adequate total sample size for two groups at the effect size *f* = 0.34/α err type = 0.05/and study power [1−*β*] err prob = 85% was 56 children.

### Recruitment and eligibility criteria

#### Inclusion criteria

1. Healthy children with no systematic diseases.
2. Children aged between 6 and 10 years old.
3. Children without previous dental history.
4. Children who recorded 2 grade [positive] on the Frankle behavior scale.
5. Children who needed dental treatment in mandibular teeth requested anesthesia with alveolar nerve block injection.
6. Children with sufficient cognitive skills to complete the self-report scale.

#### Exclusion criteria

1. Children whose parents refused to participate in this study.
2. Children with mental and physical disabilities.
3. Children with colds, asthma, and any other respiratory diseases.
4. Children with acoustic problems.
5. Children who are allergic to any of the essential oils that have been used in this study.
6. Children who took NSAIDs or analgesics drugs in the last 8 h before treatment

**Fig. 1:**
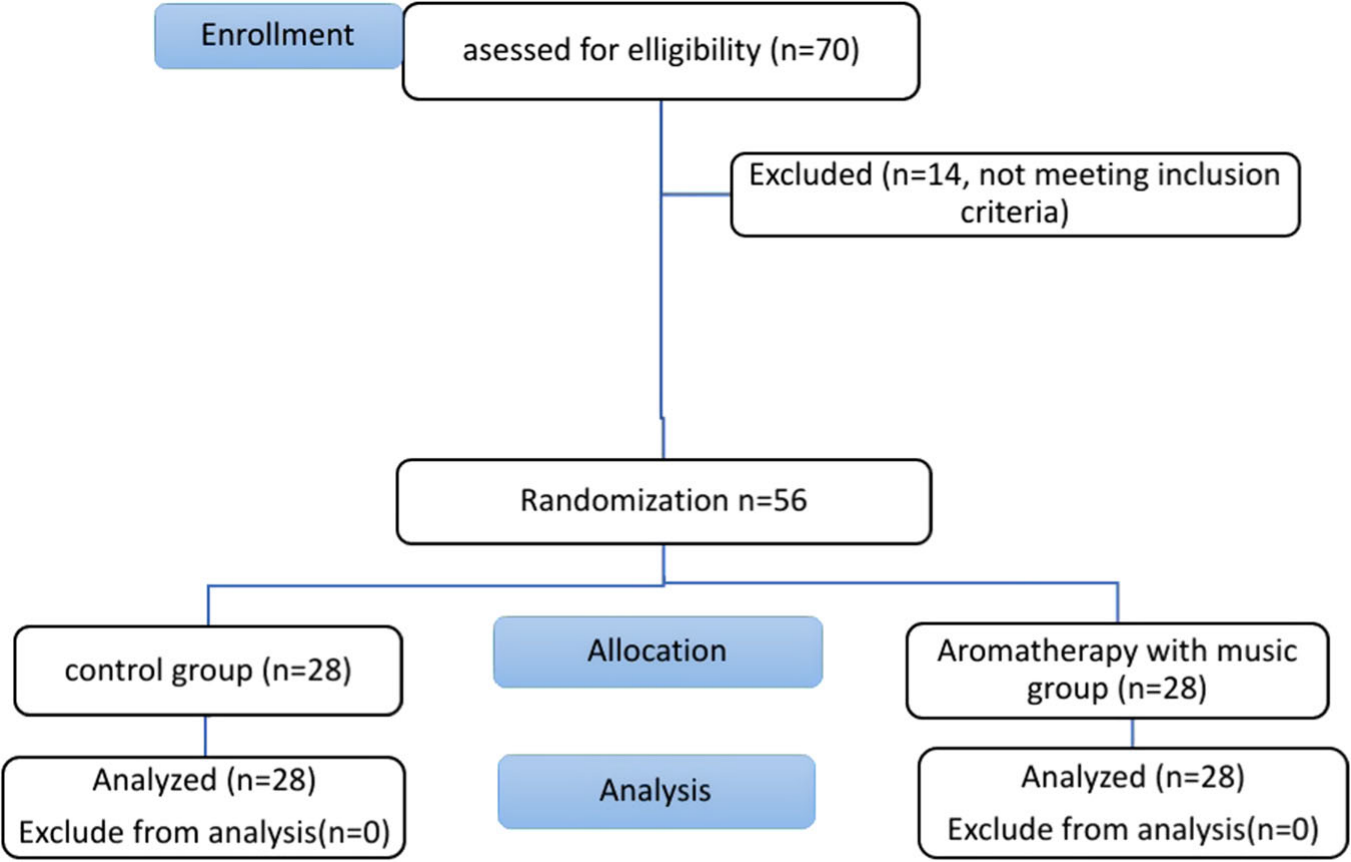
Modified CONSORT flowchart. Modified Consort flowchart.

70 children were assessed for eligibility, 14 of them were excluded due to not meeting the inclusion criteria. Finally, fifty-six children were included in this study who were randomly divided into two groups at an allocation ratio of 1:1 with a simple randomization method using the www.random.org website (Fig. 1), which was accessed on 10 October 2021. Thus, children were assigned to two groups: Group 1: control group [*n* = 28], and Group 2: aromatherapy with music group [*n* = 28].

#### Intervention

After taking demographic information and informed consent from parents, the children were randomly divided into two groups.

##### Aromatherapy combined with a music group

The Lavender-Neroli oil blend was first prepared in Damascus University’s Faculty of Pharmacology Department of Pharmacognosy by blending 2.3 ml Lavender oil [100% L. angustifolia essential oil] with 0.9 ml Neroli oil [100% C. aurantium essential oil], and the final blend was diluted into 20 ml using grape seed oil as a carrier oil. The essential oils were obtained from Biocham natural extract.co, Damascus, Syria, and the main components were determined by Gas Chromatography [GC] which found to be 37% linalool, 11.6% camphor, 9.9% 1.8 cineole, 5.5% linalyl acetate in Lavender oil, and 23.4% linalool, 15.5% linalyl acetate,12.3% trans-nerolidol, 11.9% limonene, 7.7% β pinene in Neroli oil, these data were provided by the delivering company.

**Fig. 2:**
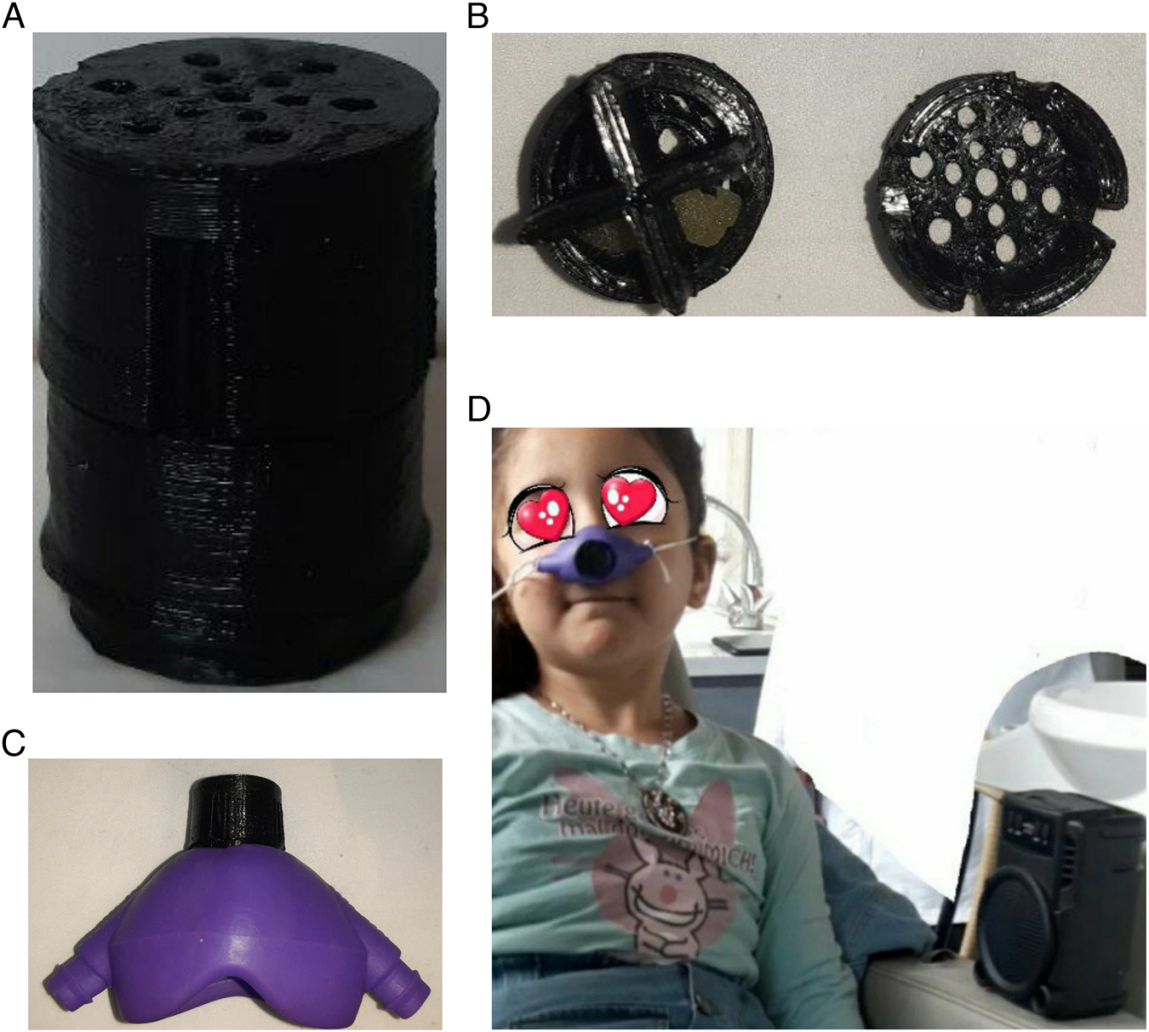
Aromatherapy and music application. Modified nitrous oxide nasal mask, (**A**) 3D printed box, (**B**) placement of the cotton balls inside box’s cavities, (**C**) the modified nasal mask after the 3D printed box was patterned on its circle hole, (**D**) A child inhale the aromatic oils from the modified nasal mask and listening to music from a speaker.

In the dental chair, the child was asked to inhale the aromatic blend and listen to self-choose music for 5 min before and during anesthesia. Aromatherapy was done by informing the child to wear a nitrous oxide nasal mask [Accturon, Hu-Friedy Mfg. Co., LLC] after modifying it by putting a 3D printed box which was perforated from its bottom and top on its circle hole, and three drops of the aromatic blend were poured on three cotton balls which were put inside the box’s cavities (Fig. 2A–D) Music in this study was done by using a wireless speaker that was connected to a mobile phone through Bluetooth and the child was asked to choose his/her favorite music from their cartoons and program on YouTube and then listen to it as a background in the treatment room (Fig. 2D).

##### Control group

Children in this study were informed to wear another modified nitrous oxide nasal mask with an empty 3D printed box was put on its circle hole as a placebo during IANB injection.

Children in both groups received IANB injections by the same research using lidocaine 2% with 1:80,000 epinephrine [Huons Lidocaine HCL, Seoul, Korea] and a 27-gauge needle [Kohope, Shanghai, China], which was used at a depth of 15 ml where 1 ml of the anesthetic’s solution was injected for 1 min [27].

### Outcomes and measurements

Dental anxiety and pain were defined as the first outcome measures and changes in physiological parameters, including heart rates, SPO2 saturation, diastolic and systolic blood pressure were considered the second.

Assessment of dental anxiety was conducted using FIS, it consisted of five faces ranging from very happy to very sad, and the child was asked to point at the face that mostly reflected his/her feelings. The score of this scale ranges from being 1 to the most positive face to being 5 to the most negative face [28, 29].

Pain assessment during anesthesia injection was done using the Arabian version of the Face-Legs -Activity-Cry-Consolability [FLACC] objective pain scale, it is considered a reliable and valid tool to assess pain in children aged 6–14 years old [30]. It includes five categories of pain behavior and each category has a score between 0 and 2, which makes the whole scale’s scores range between 0 and 10 [31].

FLACC assessment was done by using a video filmed of the child’s behavior during anesthesia and then all videos were evaluated by three trained residents in the Pediatric Dentistry Department at Damascus University who had dealt previously with the FLACC scale in their research and they were blind to the procedures in each group as the children in both groups were wearing the nasal mask with the box on it and the music sound was pulled from the video during evaluation.

physiological parameters including diastolic and systolic blood pressure, heart rate, and SPO2 saturation were assessed before and after 1 min from anesthesia injection, Heart rate and SPO2 saturation were recorded using a pulse oximeter [Meditech Equipment, Shandong, China], and diastolic and systolic blood pressure were recorded through digital blood pressure monitor with an elbow cuff [O2 medical systems, Hyderabad, India].

### Statical analysis

The data of the present study were analyzed using the SPSS 21.0 software (IBM, Armonk, NY, USA). Statistical analysis was performed using the SPSS 21.0 software (IBM, Armonk, NY, USA). The data were analyzed with Mann Whitney *U*, Wilcoxon signed-rank test, paired t-test, and Independent-T test. The testing was performed at *α* = 0.05.

## Results

Fifty-six children with a mean age of 8.2 ± 1.3 [16 boys (57.14%) and 12 girls (42.86%)] for control group, and 7.8 ± 1.2 [15 boys (53.57%) and 13 girls (46.43%)] for aromatherapy with music group had participated in this study (Table 1).

**Table 1:** Basic sample character.

Groups	Age			Gender	
	Min	Max	Means ± SD	Boys (*n* %)	Girls (*n* %)
Aromatherapy with music group	6	10	7.8 ± 1.2	15 (53.6%)	13 (46.4%)
Control group	6	10	8.2 ± 1.3	16 (57.1%)	12 (42.9%)

There were no differences in dental anxiety according to FIS scale between groups before anesthesia injection [*p* = 0.916], after anesthesia FIS score were significantly lower in aromatherapy with music group comparing to the control group [*p* = 0.000]. In a within-group, dental anxiety was increased after anesthesia injection in the control group [*p* = 0.000], whereas no significant differences were found in the aromatherapy with music group between before and after anesthesia [*p* = 1.000] (Table 2).

**Table 2:** FIS comparison between groups at different times of intervention.

Groups	FIS Baseline scores					Baseline Mean rank	FIS after anesthesia scores					After anesthesia Mean rank	*P* value^b^
	1	2	3	4	5		1	2	3	4	5		
Aromatherapy with music	18 (64.3%)	8 (28.6%)	1 (3.6%)	1 (3.6%)	0 (0%)	53.14	19 (67.9%)	6 (21.4%)	2 (7.1%)	1 (3.6%)	0 (0%)	44.63	1.000
Control	16 (57.1%)	7 (25%)	4 (14.3%)	1 (3.6%)	0 (0%)	58.34	3 (10.7%)	13 (46.4%)	5 (17.9%)	4 (14.3%)	3 (10.7%)	79.88	0.000*
*P* value^a^	-------					0.916	-------					0.000*	-------

*Significant differences at *P* < 0.05.

^a^*P* value was computed using Mann-Whitney *U* test.

^b^*P* value was computed using Wilcoxon-signed rank test.

Vital signs Including diastolic and systolic blood pressure were significantly decreased and Spo2 increased in the aromatherapy with music group after anesthesia injection [*p* = 0.000, *p* = 0.000, *p* = 0.03], respectively with no significant differences in heart rate [*p* = 0.083], while all vital signs except SPO2 saturation were significantly increased in the control group after anesthesia injection [*P* < 0.05] (Table 3). In pairwise comparison of the changes in the vital signs, there was a significant reduction in diastolic and systolic blood pressure and heart rate value in the aromatherapy with music group compared to the control [*p* = 0.000, *p* = 0.000, *p* = 0.000], respectively with no significant differences in SPO2 [*p* = 0.271] (Table 4).

**Table 3:** Vital signs comparison before and after anesthesia.

Groups	Variables	Time of intervention	Means ± SD	SE	*P* value ^a^
Aromatherapy with music	DBP	baseline	64.07 ± 17.77	3.36	0.000*
		After anesthesia	52.86 ± 16.15	3.05	
	SBP	baseline	107 ± 20.86	3.94	0.000*
		After anesthesia	93.54 ± 19.4	3.67	
	HR	baseline	95.43 ± 13.35	2.52	0.083
		After anesthesia	92.57 ± 15.06	2.85	
	SPO2	baseline	97.39 ± 1.77	0.33	0.03*
		After anesthesia	98.29 ± 1.12	0.21	
Control	DBP	Baseline	59.57 ± 11.59	2.19	0.024*
		After anesthesia	66.61 ± 16.31	3.08	
	SBP	baseline	96.36 ± 9.97	1.88	0.004*
		After anesthesia	102.61 ± 11.33	2.14	
	HR	baseline	92.79 ± 15.76	2.98	0.000*
		After anesthesia	101.04 ± 19.34	3.67	
	SPO2	baseline	97.93 ± 1.09	0.21	0.485
		After anesthesia	98.11 ± 1.13	0.21	

*DBP* diastolic blood pressure, *SBP* systolic blood pressure, *HR* heart rate, *SPO2* oxygen saturation, *SD* standard deviation, *SE* standard error.

*Significant differences at *P* < 0.05.

^a^*P* value was computed using paired *t* test.

**Table 4:** Comparison of changes in vital signs value [after vs baseline] between groups.

Variables	Groups	Means ± SD	SE	*P* value^a^
ΔDBP	Aromatherapy with music group	−11.21 ± 12.2	2.94	0.000*
	Control group	7.04 ± 15.57	2.31	
ΔSBP	Aromatherapy with music group	−13.46 ± 10.46	2.88	0.000*
	Control group	6.25 ± 15.25	1.98	
ΔHR	Aromatherapy with music group	−2.86 ± 10.17	1.59	0.000*
	Control group	8.25 ± 8.4	1.92	
ΔSPO2	Aromatherapy with music group	0.89 ± 2.06	0.39	0.271
	Control group	0.18 ± 1.33	0.25	

*Δ* Changes in value after vs baseline.

*Significant differences at *P* < 0.05.

^a^*P* value was computed using independent *t* test.

There are no significant differences between groups in pain perception according to FLACC scale [*p* = 0.176] (Table 5).

**Table 5:** Pairwise comparison in FLACC Value during anesthesia.

Groups	Means ± SD	SE	*P* value
Aromatherapy with music group	1.83 ± 1.99	0.38	0.176*
Control group	3.11 ± 2.42	0.46	

^*^Significant differences at *P* < 0.05.

## Discussion

Management of children’s dental fear is a basic practice for pediatric dentists, as it plays the main role in preventing children from having appropriate oral health care [32]. Fear of injection and painful IANB injection is one of the most common reasons for children refusing dental treatment which can badly affect the child’s behavior [27].

In this study, aromatherapy with a blend of Lavender-Neroli oil combined with music two non-pharmacological techniques that were used to investigate their efficacy in reducing pediatric dental anxiety and pain during IANB injection. Essential oils were chosen due to the effectiveness of the citrus and Lavender essential oils in managing anxiety [33, 34]. Furthermore, the acceptance of the mixture’s smell is important for obtaining the relaxation effect of aromatherapy [35]. To ensure the acceptance of the final blending scent, 20 children were been asked about how pleasant is the odor before starting the study. Music in this study was chosen by children from their cartoons which includes different music’s genres such as classic, pop, and other. Music’s characteristics, such as melody, harmony, and rhythm play an important role on the listener’s emotions. However, the effect of music on the psychological aspects is more individual and differs from one person to another for the same piece of music due to its links to memories, culture, and experience which does not make for example relaxation music always effective [25]. Using self-chosen music reduces the effect of bad memories, and increases the interaction with it which helps to induce pleasure and distract the child’s attention.

Listening to self-chosen music also reduces the fear of the unknown and makes the dental environment more familiar to the child [36]. Al-Halabi et al. [37] indicated that techniques that block the outer environment could increase the child’s anxiety from the expected danger in dental procedures [37]. In the current study, Music was played as background in the treatment room which has an advantage over using headphones as it keeps the communication between the child and the dentist [36].

Anxiety is a complex phenomenon that needs more comprehensive assessment tools which leads us to more understanding of the patient’s feelings [38]. Self-report anxiety scales are the simplest and most common way to ask children about their feelings [2].

In this study, the FIS pictorial self-report anxiety scale was used due to its validation and ease of use in children with young age and low cognitive skills [28]. It only requires good communication skills that 6-year-old child could have with no need for writing and reading skills. One of the methods to measure dental anxiety is to investigate its effect on the physiological aspects. Anxiety can arousal the stress hormones and nervous system which activate the circulation and respiratory system [39]. In the current study, changes in heart rate, diastolic and systolic blood pressure, and SPO2 were used as a physiological scale.

In this study, aromatherapy with Lavender-Neroli oil combined with music was effective in reducing dental anxiety and physiological parameters except SPO2 saturation compared to the control group. Sensory such as olfactory, auditory, and visual have inputs in the amygdala the part of the limbic system where emotions such as anxiety are developed [16]. Music induces an anxiolytic effect by suppressing the amygdala and releasing endorphins, such as dopamine in the limbic system [36, 40].

Aromatic molecules also affect the limbic system and amygdala, which responds to olfactory stimulus by releasing anxiolytic neurotransmitters such as serotonin and endorphins, which are happiness hormones that improve mood and induce calmness [11, 16]. In addition, the components in essential oils play a pharmacological role in reducing anxiety. Gas chromatography has shown that the main component of the oils that have been used in the blend is linalool. Linalool is a terpenoid that affects the mechanism that regulars the central nerve system activation. Linalool inhibits glutamatergic receptors and binds to GABA receptors similarly to benzodiazepines which provide a sedative effect in CNS [22, 41].

Other anxiolytic components were found in a lower concentration such as linalyl acetate which has an impact on behavior, and limonene which suppress releasing stress hormone such as cortisol along HPA axes [11, 42]. Children in both groups were informed to wear a nasal mask which affects the SPO2 saturation, wearing a mask can affect normal breathing due to the increasing humidity and CO2 in the dead space of the mask [43].

There is a correlation between pain and anxiety [44]. FLACC objective pain scale was used to assess the pain during needle injection. In this study, there are no differences between groups in pain perception. This could be due to the short duration of these procedures, also all children in both groups were informed to wear a nasal mask which affects the pain perception as it distracts attention from seeing the needle.

The results of this study were in accordance with the results of Pradopo et al. [26] and Janthasila et al. [9] as they revealed the effectiveness of aromatherapy combined with music in reducing dental anxiety in children during prophylaxis dental procedures [9, 26]. In contrast to this study, Rohi Ganji et al. [40] found that the combination of the two techniques wasn’t effective in reducing anxiety during shock wave lithotripsy in adult patients, this could be due that music hasn’t been the patients’ choice and the stressful type of the procedure which make the patient more anxious [40].

In this study, aromatherapy was safe and no adverse effect noted. In general, essential oils are safe plant metabolisms with minimal side effects including skin, eye, and mucous membrane irritation and sensitivity due to topical application [11]. Essential oils that are applied topical should be diluted in vegetable oils to avoid irritation [14]. The inhalation method is the most widely used in aromatherapy due to the simplest of administrations [19, 34].

In this study, to make aromatherapy more safe aromatic oils were diluted in grape seed oil, grape seed oil is one of the vegetable oils that are considered odorless and plays a base oil role to ensure the harmony of the oil mixture [45]. Furthermore, aromatherapy in this study was delivered through an external 3D printed box which was put in the circle hole on the top of the nitrous oxide nasal mask that makes the odor from aromatic oils easy to remove and suppling oxygen in the case of respiratory sensitives could happen.

The strength of this study was the use of both psychological and physiological anxiety scales along with pain assessment, and the use of gas chromatography which determines the active components in essential oils. However, there are some limitations in this study, no control odor was used as the effect of aromatherapy could be due to the change of clinic sent, also the procedures were limited to anesthesia and did not study the consciously effect of the techniques on long procedures such as restorative treatment, another limitation is the long period of the study which requires ensuring the validity of the blending oils, which was done by preparing a fresh blending every 2 weeks and keeping it in good conditions at a temperature of 21° and away from light so that its physical properties are not affected.

## Conclusion

The results of this study conclude that aromatherapy combined with music can be considered an effective, low-cost, simple, and safe technique for managing dental anxiety in children. Further study should be done to confirm their effect with different essential oils and music genres through different dental procedures.

## Data Availability

All data related to this research are mentioned in this paper.

## References

[1] Alshuaibi AF, Aldarwish M, Almulhim AN, Lele GS, Sanikommu S, Raghunath RG (2021). Prevalence of dental fear and anxiety and its triggering factors in the dental office among school-going children in Al Ahsa. Int J Clin Pediatr Dent.

[2] Yon MJY, Chen KJ, Gao SS, Duangthip D, Lo ECM, Chu CH (2020). An introduction to assessing dental fear and anxiety in children. Healthcare.

[3] Grisolia BM, dos Santos APP, Dhyppolito IM, Buchanan H, Hill K, Oliveira BH (2021). Prevalence of dental anxiety in children and adolescents globally: a systematic review with meta-analyses. Int J Paediatr Dent.

[4] Cianetti S, Lombardo G, Lupatelli E, Pagano S, Abraha I, Montedori A (2017). Dental fear/anxiety among children and adolescents. a systematic review. Eur J Paediatr Dent.

[5] Avramova N (2022). Dental fear, anxiety, and phobia; causes, diagnostic criteria and the medical and social impact. J Mind Med Sci.

[6] Carrillo-Díaz M, Migueláñez-Medrán BC, Nieto-Moraleda C, Romero-Maroto M, González-Olmo MJ (2021). How can we reduce dental fear in children? The importance of the first dental visit. Children.

[7] Karan NB (2019). Influence of lavender oil inhalation on vital signs and anxiety: a randomized clinical trial. Physiol Behav.

[8] Appukuttan DP (2016). Strategies to manage patients with dental anxiety and dental phobia: literature review. Clin Cosmet Investig Dent [Internet].

[9] Janthasila N, Keeratisiroj O (2023). Music therapy and aromatherapy on dental anxiety and fear: a randomized controlled trial. J Dent Sci.

[10] Lizarraga-Valderrama LR. Effects of essential oils on central nervous system: focus on mental health. Phytother Res. 2021;35:657–79. 10.1002/ptr.6854 32860651

[11] Ali B, Al-Wabel NA, Shams S, Ahamad A, Khan SA, Anwar F (2015). Essential oils used in aromatherapy: a systemic review. Asian Pac J Trop Biomed.

[12] Purohit A, Singh A, Purohit B, Shakti P, Shah N (2021). Is aromatherapy associated with patient’s dental anxiety levels? A systematic review and meta-analysis. J Dent Anesth Pain Med.

[13] Bikmoradi A, Khaleghverdi M, Seddighi I, Moradkhani S, Soltanian A, Cheraghi F (2017). Effect of inhalation aromatherapy with lavender essence on pain associated with intravenous catheter insertion in preschool children: a quasi-experimental study. Complement Ther Clin Pract.

[14] Reyes MCGM, Reyes MCGM, Ribay KGL, Paragas ED (2020). Effects of sweet orange aromatherapy on pain and anxiety during needle insertion among patients undergoing hemodialysis: a quasi-experimental study. Nurs Forum [Internet].

[15] Arslan I, Aydinoglu S, Karan NB (2020). Can lavender oil inhalation help to overcome dental anxiety and pain in children? A randomized clinical trial. Eur J Pediatr.

[16] Alkanan SAM, Alhaweri HS, Khalifa GA, Ata SMS (2023). Dental pain perception and emotional changes: on the relationship between dental anxiety and olfaction. BMC Oral Health [Internet].

[17] Nirmala K, Kamatham R (2021). Effect of aromatherapy on dental anxiety and pain in children undergoing local anesthetic administrations: a randomized clinical trial. J Caring Sci.

[18] Naufal AH, Virgirinia RP, Fatchiyah F (2023). Molecular interaction of lavender [Lavandula angustifolia Mill] essential oil compounds as potential anxiolytic against α2δ subunit voltage gated calcium channel. Berk Penelit Hayati..

[19] Donelli D, Antonelli M, Bellinazzi C, Gensini GF, Firenzuoli F (2019). Effects of lavender on anxiety: a systematic review and meta-analysis. Phytomedicine.

[20] Moslemi F, Alijaniha F, Naseri M, Kazemnejad A, Charkhkar M, Heidari MR (2019). Citrus aurantium aroma for anxiety in patients with acute coronary syndrome: a double-blind placebo-controlled trial. J Altern Complement Med.

[21] Scandurra C, Mezzalira S, Cutillo S, Zapparella R, Statti G, Maldonato NM (2022). The effectiveness of neroli essential oil in relieving anxiety and perceived pain in women during labor: a randomized controlled trial. Healthcare.

[22] Borba CA, Fernandes GV, Campos JC, Silva TBda, Gonzaga RV (2021). Potential action on the central nervous system of neroli oil extracted from Citrus aurantium. Res Soc Dev.

[23] Mejía-Rubalcava C, Alanís-Tavira J, Mendieta-Zerón H, Sánchez-Pérez L (2015). Changes induced by music therapy to physiologic parameters in patients with dental anxiety. Complement Ther Clin Pract.

[24] Aravena PC, Almonacid C, Mancilla MI. Effect of music at 432 Hz and 440 Hz on dental anxiety and salivary cortisol levels in patients undergoing tooth extraction: a randomized clinical trial. J Appl Oral Sci. 2020;28:e20190601. 10.1590/1678-7757-2019-0601. 10.1590/1678-7757-2019-0601 PMC7213780 32401941

[25] Mahmood D, Nisar H, Yap VV, Tsai CY (2022). The effect of music listening on EEG functional connectivity of brain: a short-duration and long-duration study. Mathematics.

[26] Pradopo S, Sinaredi BR, Januarisca BV (2017). Pandan Leaves [Pandanus Amaryllifolius] aromatherapy and relaxation music to reduce dental anxiety of pediatric patients. J Int Dent Med Res.

[27] Dean JA. Mcdonald and avery’s dentistry for the child and adolescent. 11th Edition. Dean JA, editor. *Elsevier Health Science*; 2021. p 326–330.

[28] Fathima F, Jeevanandan G (2018). Validation of a facial image scale to assess child dental anxiety. Drug Invent Today.

[29] Tiwari S, Kulkarni P, Agrawal N, Mali S, Kale S, Jaiswal N (2021). Dental anxiety scales used in pediatric dentistry: a systematic review and meta-analysis. J Contemp Dent Pract.

[30] Dak Albab R, Shakhashero H. The validity and reliability of the Arabic version of FLACC scale: a clinical trial. J Anesth Clin Res. 2016;7:656. 10.4172/2155-6148.1000656.

[31] Crellin DJ, Harrison D, Santamaria N, Huque H, Babl FE (2018). The psychometric properties of the FLACC scale used to assess procedural pain. J Pain.

[32] Shukla H, Kulkarni S, Wasnik MB, Rojekar N, Bhattad D, Kolekar P (2021). Acceptance of parents for behavior management technique with reference to previous dental expertise and dental anxiety. Int J Clin Pediatr Dent.

[33] Cai H, Xi P, Zhong L, Chen J, Liang X. Efficacy of aromatherapy on dental anxiety: a systematic review of randomised and quasi-randomised controlled trials. Oral Dis. 2021;27:829–47. 10.1111/odi.13346 32267044

[34] Guo P, Li P, Zhang X, Liu N, Wang J, Yang S (2020). The effectiveness of aromatherapy on preoperative anxiety in adults: a systematic review and meta-analysis of randomized controlled trials. Int J Nurs Stud.

[35] Siripornpanich V, Kotchabhakdi N (2012). The effects of lavender oil inhalation on emotional states, autonomic nervous system, and brain electrical activity sport neurophysiology view project role of melatonin in neuronal differentiation view project. Artic J Med Assoc Thail Chotmaihet Thangphaet [Internet].

[36] Bradt J, Teague A. Music interventions for dental anxiety. Oral Dis. 2018;24:300–6. 10.1111/odi.12615 27886431

[37] Al-Halabi MN, Bshara N, AlNerabieah Z (2018). Effectiveness of audio visual distraction using virtual reality eyeglasses versus tablet device in child behavioral management during inferior alveolar nerve block. Anaesth Pain Intensive Care..

[38] Shindova MP, Belcheva AB (2021). Dental fear and anxiety in children: a review of the environmental factors. Folia Med.

[39] Ainscough SL, Windsor L, Tahmassebi JF (2018). A review of the effect of music on dental anxiety in children. Eur Arch Paediatr Dent.

[40] Rohi Ganji M, Jafari F, Rezaeian S, Abdi H, Farzaei MH, Khatony A. The effect of inhalation aromatherapy and music therapy on anxiety in patients undergoing shockwave lithotripsy: a randomized controlled clinical trial. Evid-Based Complement Altern Med. 2022. 10.1155/2022/8015798. 10.1155/2022/8015798 PMC9259239 35815287

[41] Agatonovic-Kustrin S, Kustrin E, Gegechkori V, Morton DW. Anxiolytic terpenoids and aromatherapy for anxiety and depression. Adv Exp Med Biol. 2020;1260:283–96. 10.1007/978-3-030-42667-5_11. 10.1007/978-3-030-42667-5_11 32304038

[42] Eddin LB, Jha NK, Meeran MFN, Kesari KK, Beiram R, Ojha S (2021). Neuroprotective potential of limonene and limonene containing natural products. Molecules.

[43] Nwosu ADG, Ossai EN, Onwuasoigwe O, Ahaotu F (2021). Oxygen saturation and perceived discomfort with face mask types, in the era of COVID-19: a hospital-based cross-sectional study. Pan Afr Med J..

[44] Wajda M, Gover A, Franco L, Blanck T. Review of lavender aromatherapy: past, present, and future. Austin Ther [Internet]. 2017;4:1029. www.austinpublishinggroup.com.

[45] Michalak M (2018). The use of carrier oils in aromatherapy massage and their effect on skin. Arch Physiother Glob Res..

